# Estimating the number of people with hepatitis C virus who have ever injected drugs and have yet to be diagnosed: an evidence synthesis approach for Scotland

**DOI:** 10.1111/add.12948

**Published:** 2015-06-08

**Authors:** Teresa C. Prevost, Anne M. Presanis, Avril Taylor, David J. Goldberg, Sharon J. Hutchinson, Daniela De Angelis

**Affiliations:** ^1^ MRC Biostatistics UnitCambridge Institute of Public HealthCambridgeUK; ^2^ School of Culture, Media and SocietyUniversity of the West of ScotlandPaisleyUK; ^3^ Health Protection ScotlandNHS National Services ScotlandGlasgowUK; ^4^ School of Health and Life SciencesGlasgow Caledonian UniversityGlasgowUK

**Keywords:** Evidence synthesis, hepatitis C, people who inject drugs, prevalence

## Abstract

**Aims:**

To estimate the number of people who have ever injected drugs (defined here as PWID) living in Scotland in 2009 who have been infected with the hepatitis C virus (HCV) and to quantify and characterize the population remaining undiagnosed.

**Methods:**

Information from routine surveillance (*n* = 22 616) and survey data (*n* = 2511) was combined using a multiparameter evidence synthesis approach to estimate the size of the PWID population, HCV antibody prevalence and the proportion of HCV antibody prevalent cases who have been diagnosed, in subgroups defined by recency of injecting (in the last year or not), age (15–34 and 35–64 years), gender and region of residence (Greater Glasgow and Clyde and the rest of Scotland).

**Results:**

HCV antibody‐prevalence among PWID in Scotland during 2009 was estimated to be 57% [95% CI=52−61%], corresponding to 46 657 [95% credible interval (CI) = 33 812–66 803] prevalent cases. Of these, 27 434 (95% CI = 14 636–47 564) were undiagnosed, representing 59% [95% CI=43−71%] of prevalent cases. Among the undiagnosed, 83% (95% CI = 75–89%) were PWID who had not injected in the last year and 71% (95% CI = 58–85%) were aged 35–64 years.

**Conclusions:**

The number of undiagnosed hepatitis C virus‐infected cases in Scotland appears to be particularly high among those who have injected drugs more than 1 year ago and are more than 35 years old.

## Introduction

Hepatitis C virus (HCV) is a major cause of chronic liver disease, leading potentially to cirrhosis and hepatocellular carcinoma 1. The greatest risk of HCV infection in resource‐rich countries comes from injecting drug use 2. With an estimated 16 million people world‐wide currently injecting drugs 3, 10 million of whom have already been infected, in this population HCV represents a significant global public health challenge 2.

As spontaneous viral clearance occurs in only approximately 25% of those diagnosed HCV‐antibody‐positive 4, effective treatment strategies are crucial in reducing the demand on health‐care systems from chronic HCV. The development of more effective antiviral therapies—with reduced toxicity, simplified oral dosing and shortened regimens—will majorly transform the treatment of HCV infection in future 5. For these new therapies to have any great impact on the burden of HCV, particularly among people who inject drugs (PWID) 6, effective targeting of HCV screening and case‐finding initiatives is essential. To achieve this, understanding the size and characteristics of the infected populations, involving not just diagnosed individuals, but importantly those remaining undiagnosed, is crucial. Reliable estimation of these quantities is not straightforward, as direct data are not readily available. Instead, we rely upon a multiplicity of information, typically related indirectly to the quantities of interest.

Scotland has an extensive national HCV surveillance programme established to inform and monitor the impact of its Government Action Plan 7. A wealth of epidemiological data on the PWID and HCV‐diagnosed populations is available, more than in most other countries, which may be exploited usefully in a multiparameter evidence synthesis (MPES) to estimate anti‐HCV antibody prevalence (HCV prevalence hereafter). MPES combines direct and indirect information, accounting for uncertainty in and potentially resolving any inconsistencies between data sources 8, 9, 10, 11, 12, 13.

A Bayesian approach to MPES was applied here to:

- estimate the number of PWID living in Scotland who are HCV‐prevalent in 2009, and
- quantify and characterize the infected PWID population remaining undiagnosed.

In addition, the MPES approach enabled estimation of the total number of PWID; namely, all those who have ever injected, even though no directly relevant data were available, due to the inherent difficulties surveying this risk group.

## Methods

The analysis proceeded in two stages. In stage 1, the following estimates were obtained:

1.1 *Number of HCV‐diagnosed PWID*, estimated from the linkage of the Scottish Drugs Misuse Database (TrtDat) 14 to the Scottish Hepatitis C Diagnosis Database (DiagDat) 15. TrtDat records attendance at drug treatment services, whereas DiagDat records HCV diagnoses.
1.2 *Number of HCV‐diagnosed recently injecting PWID*, using data from TrtDat to predict whether HCV‐diagnosed PWID had injected recently.

Note that ‘recently’ is defined as having injected in the last year (see Discussion for further consideration of this definition).

In stage 2, estimates of the size of the non‐recently injecting PWID population and both the total and undiagnosed HCV‐infected PWID populations were derived. This involved combining information on:

2.1 *Size of the recently injecting PWID population* from a capture–recapture (CR) study 16
2.2 *HCV prevalence in recently and non‐recently injecting PWID and proportion that are diagnosed* from a Needle Exchange Surveillance Initiative (NSP) 17, 18
2.3 *Number of HCV‐diagnosed recently and non‐recently injecting PWID* from stage 1.

### Bayesian MPES framework

Throughout we adopted a Bayesian framework for estimation 19. This approach consists of:

- Defining prior distributions: before looking at the data, anything known about the basic parameters (e.g. HCV prevalence) is expressed as a probability distribution (the prior distribution). This is flat, with equal probability across all possible values, when no specific information is available or peaked otherwise (e.g. if evidence is available from a previous study).
- Relating data to parameters: the observed data are assumed to be realizations from a distribution (see Model details below) and used to construct a ‘likelihood’ function, which describes the relationship between the data and the basic parameters, quantifying the support that the data provide to the possible parameter values.
- Obtaining posterior distributions: the prior distribution is updated with the information from the data likelihood to form a posterior distribution, combining from both prior knowledge and data. In principle, this distribution is proportional to the product of the prior and the likelihood. For complex models, however, an analytical expression for this distribution cannot be derived easily. Instead, we simulate from the posterior distribution using a Markov chain Monte Carlo algorithm 20. We use the posterior samples of the basic parameters to estimate the key quantities of interest. All posteriors are summarized in terms of posterior medians and 95% credible intervals (CI).

A Bayesian MPES approach incorporates data from multiple sources, potentially including information known to be affected by biases, which then are modelled explicitly. The Bayesian approach was implemented in OpenBUGS 21, with posterior estimates for all parameters of interest based on 100 000 samples.

### Epidemiological model

As HCV prevalence can vary over time and depends upon demographic characteristics among PWID, we estimated the size of the HCV‐infected population according to: (a) recency of injecting [recent (*R*) and non‐recent (*NR*)], (b) age group (15–34 and 35–64 years), (c) gender and (d) region of residence [Greater Glasgow and Clyde (Glasgow) and the rest of Scotland]*.* Denoting by *i* the recency of injecting, *i* ∈ {*R*, *NR*} and *d* the demographic groups defined by age (*a*), gender (*g*) and region (*r*), such that *d* = {*a*, *g*, *r*}, define:

- *ρ_i,d_*, the proportion of the population in demographic group *d* with recency of injecting *i*;
- *π_i,d_*, the HCV prevalence in subgroup {*i,d*};
- δ*_i,d_*, the proportion of HCV‐infected cases in subgroup {*i,d*} that are diagnosed;
- *T_d_*, the size of demographic group *d* in the general population.

### Stage 1: Estimating the number of HCV‐diagnosed recent and non‐recent PWID

For each demographic group *d*, the following estimates were obtained.

**Figure 1:**
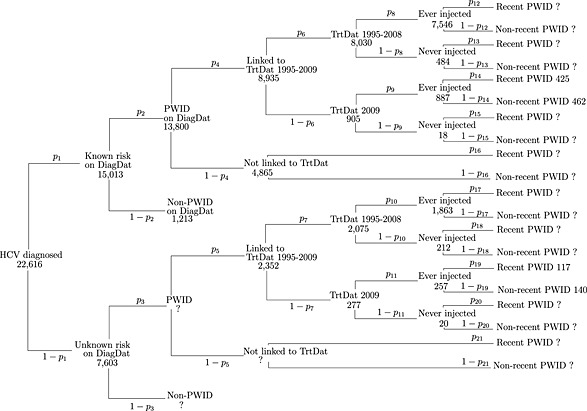
Individuals aged 15–64 years diagnosed with hepatitis C virus (HCV) in Scotland by the end of 2009 (and not known to have died) by risk group, as recorded on the Hepatitis C Diagnosis Database (DiagDat) and linked Drugs Misuse Database (TrtDat) data. Recent and non‐recent PWID (people who inject drugs) refers to status in 2009

1.1 HCV‐diagnosed PWID *T*_*d*_ (*ρ*_*R*,*d*_ *π*_*R*,*d*_ *δ*_*R*,*d*_ + *ρ*_*NR*,*d*_ *π*_*NR*,*d*_ *δ*_*NR*,*d*_)
  Since 1991 the DiagDat has recorded information on all individuals diagnosed HCV‐positive in Scotland 15. Of the 22 616 individuals aged 15–64 years recorded by the end of 2009 (and not known to have died by mid‐2009), the risk factor for HCV at time of diagnosis was injecting drug use for 61%, other risk factor (e.g. blood transfusion) for 5% and unknown for 34%. Some diagnosed individuals with unknown risk were identified as being PWID through linkage of DiagDat with TrtDat (*n* = 2352), which contains data on those who had attended drug‐treatment services since April 1995 14. Of the remainder with unknown risk, the proportion who were PWID was estimated based on the observed proportion and the model in Fig. 1. Figure 1 shows the data structure of DiagDat linked to TrtDat, where HCV‐diagnosed individuals are subdivided into recent PWID, non‐recent PWID and non‐PWID in 2009. The parameters *p*_*j*_ (*j* = 1, …, 21) denote the probabilities of possible subdivisions at each branching.
  For example, *p_3_* represents the probability that an HCV‐diagnosed individual with unknown risk group at diagnosis is a PWID; *p_12_* represents the probability that an HCV‐diagnosed individual, with PWID risk at diagnosis and ever‐injector status in TrtDat in 1995–2008, was a recent PWID in 2009; and *p_17_* represents the probability that an HCV‐diagnosed individual, with unknown risk group at diagnosis and ever‐injector status in TrtDat in 1995–2008, was a recent PWID in 2009.
1.2 HCV‐diagnosed recent PWID *T*_*d*_ *ρ*_*R*,*d*_ *π*_*R*,*d*_ *δ*_*R*,*d*_
  While the information held on DiagDat cannot distinguish between a recent and non‐recent PWID, TrtDat records whether an individual injected in the last month. However, this can only be considered to reflect recent behaviour in those last registered with a drug service in 2009. For those last registered prior to 2009, a prediction of their recent/non‐recent PWID status in 2009 was made based on individual characteristics relating to injecting behaviour using a regression approach (see Supporting information, Appendix S1 for details).

In Fig. 1, the number of individuals at each branching, *y*_*j*_ (*j* = 1, …, 21), was assumed to be a realization from a binomial distribution with unknown probability, *p_j_*, and denominator equal to its ‘parent’, *n_j_*, such that *y*_*j*_ ∼ Binomial(*n*_*j*_, *p*_*j*_). For example, the number of PWID in the unknown risk group is assumed to be from a binomial distribution with probability *p_3_* and denominator equal to the number in the unknown risk group (*n*_3_ = 7603). To identify the total number of recent and non‐recent PWID, it was necessary to constrain some of the unknown probability parameters. Table 1 gives details of these constraints and the prior distributions employed in the model.

**Table 1:** Prior assumptions for the parameters in the stage 1 model.

Parameter	Prior assumption	Comment
*p_j_*	Uniform (0,1)	Flat prior distribution
(for *j = 1,2,4,…,11*)		
*p_3_*	Uniform (0.6, *p_2_*)	The prevalence of PWID in Scotland's HCV‐diagnosed has been estimated as 83% (95% CI = 81–87%) 22, which would imply that 18 771 of the 22 616 diagnosed are PWID. 13 800 were known PWID from DiagDat, leaving 4971 unknown PWID. This gives the probability that a diagnosed individual with unknown risk was a PWID as 0.65 (95% CI = 59–77%)
prob(PWID | unknown risk group at diagnosis)		
*p_13_*	*p_13_* = *p_12_*	The probability that a known PWID ‘never injector’, linked to TrtDat in 1995–2008, had recently injected was assumed to be equal to that for a known PWID ‘ever injector’ linked to TrtDat in 1995–2008
prob(recent injector 2009 | PWID risk group at diagnosis and never injector in TrtDat 1995–2008)		
*p_16_*	*p_16_ ~* Uniform (0, *p_14_*)	The probability that a known PWID, not linked to TrtDat, had recently injected was assumed to be lower than that for a known PWID ‘ever injector’ who linked to TrtDat in 2009
prob(recent injector 2009 | PWID risk group at diagnosis and not in TrtDat)		
*p_15_*	*p_15_* = *p_14_*	The probability that a known PWID ‘never injector’, linked to TrtDat in 2009, had recently injected was assumed to be equal to that for a known PWID ‘ever injector’ linked to TrtDat in 2009
prob(recent injector 2009 | PWID risk group at diagnosis and never injector in TrtDat 2009)		
*p_18_*	*p_18_* = *p_17_*	The probability that an unknown PWID ‘never injector’, linked to TrtDat in 1995–2008, had recently injected was assumed to be equal to that for an unknown PWID ‘ever injector’ linked to TrtDat in 1995–2008
prob(recent injector 2009 | unknown PWID risk group at diagnosis and never injector in TrtDat 1995–2008)		
*p_21_*	*p_21_ ~* Uniform (0, *p_19_*)	The probability that an unknown PWID, not linked to TrtDat, had recently injected was assumed to be lower than that for an unknown PWID ‘ever injector’ who linked to TrtDat in 2009
prob(recent injector 2009 | unknown PWID risk group at diagnosis and not in TrtDat)		
*p_20_*	*p_20_* = *p_19_*	The probability that an unknown PWID ‘never injector’, linked to TrtDat in 2009, had recently injected was assumed to be equal to that for an unknown PWID ‘ever injector’ linked to TrtDat in 2009
prob(recent injector 2009 | unknown PWID risk group at diagnosis and never injector in TrtDat 2009)		

CI = confidence interval; PWID = people who inject drugs; DiagDat = Hepatitis C Diagnosis Database; TrtDat = Drugs Misuse Database.

Inference about the parameters in the regression model and the *p*_*j*_ (*j* = 1, …, 21) were made simultaneously, providing estimates of the number of diagnosed PWID and diagnosed recent PWID in each demographic group (OpenBUGS model code in Supporting information, Appendix S6).

### Stage 2: Estimating the number of HCV‐infected recent and non‐recent PWID and the number undiagnosed

**Figure 2:**
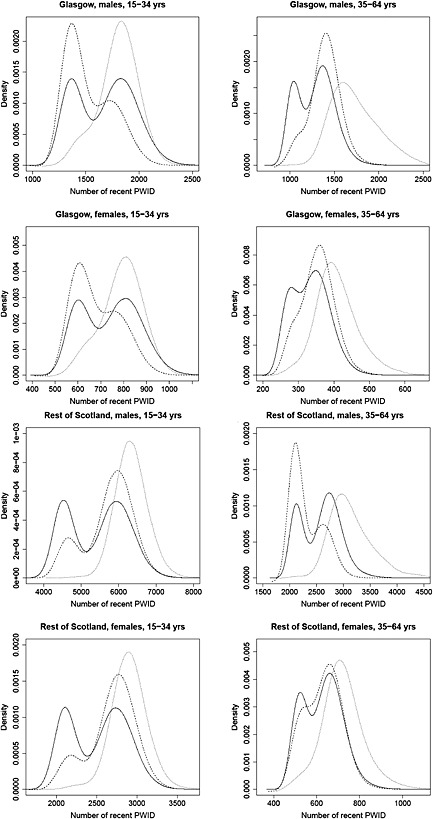
Capture–recapture (CR) estimates of the number of recent people who inject drugs (PWID) used as prior distribution for T_d_ *ρ_R,d_* in evidence synthesis model and posterior distributions for size of the recent PWID population as estimated by evidence synthesis models, with and without bias adjustment parameters, by region of residence, gender and age. 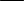 CR prior distribution, 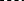 posterior distribution for model with bias adjustment parameters (baseline model), 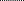 posterior distribution for model without bias adjustment parameters (sensitivity 2)

The following estimates for each demographic group *d* were combined using MPES:

2.1 Number of recently‐injecting PWID *T*_*d*_ *ρ*_*R*,*d*_
  The CR study 16 generated estimates (Supporting information, Appendix S2) of the number of current PWID in Scotland in 2009 by age, gender and region, which provide information on the size of the recent PWID population via a prior distribution. Note that this prior is bimodal (Fig. 2 and Supporting information, Appendix S2), as the CR results were obtained by averaging estimates over different models 16.
2.2 HCV prevalence in recent and non‐recent PWID (*π*_*R*,*d*_, *π*_*NR*,*d*_) and proportion diagnosed (*δ*_*R*,*d*_, *δ*_*NR*,*d*_)
  NSP is a voluntary anonymous survey of PWID, conducted nationally at approximately 100 selected needle exchange services 17, 18. Participants provide a blood‐spot sample for HCV testing and information on any previous HCV diagnosis. From the 2008–09 survey, data on HCV prevalence in PWID (*n* = 2511), both recent (*n* = 1738) and non‐recent (*n* = 772), and on the diagnosed proportion in these groups were available (Supporting information, Appendix S2). A recent PWID was defined in NSP by injection in the last month: a sensitivity analysis using injection in the last 6 months instead found the main results unchanged. NSP participants attend services providing injecting equipment and other harm‐reduction services and so are potentially more likely to have been tested for HCV than those not attending these services, which could result in an overestimate of the proportion diagnosed. This potential bias has been modelled explicitly by including an additional unknown age‐specific bias parameter, $b_{a}^{\delta }$, representing the log odds ratio of the NSP estimated relative to the ‘true’ diagnosed proportion (Supporting information, Appendix S3).
2.3 Number of HCV‐diagnosed PWID, *T*_*d*_ (*ρ*_*R*,*d*_ *π*_*R*,*d*_ *δ*_*R*,*d*_ + *ρ*_*NR*,*d*_ *π*_*NR*,*d*_ *δ*_*NR*,*d*_) and HCV‐diagnosed recent PWID, *T*_*d*_ *ρ*_*R*,*d*_ *π*_*R*,*d*_ *δ*_*R*,*d*_ (estimated in stage 1)
  TrtDat records data at the first attendance in at least 6 months to a particular drug treatment service. This source is thus probably biased towards recent, rather than non‐recent, PWID, generating an overestimate of the number of diagnosed recent PWID from stage 1. The stage 2 model includes an additional age‐specific parameter to account for this potential bias, $b_{a}^{D}$, representing the ratio of the TrtDat estimated to the ‘true’ number of diagnosed recent PWID (Supporting information, Appendix S3).

### Model details

**Figure 3:**
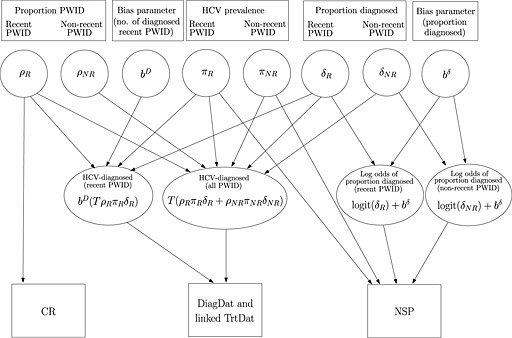
Relationship between the parameters and the data sources. Circles denote the unknown parameters (or functions of parameters) which are to be estimated. Squares denote the data sources. A link between a parameter (or function of parameters) and a data source indicates that the data source provides information on that parameter (or function of parameters). *ρ* : proportion of the population in risk group; *π* : hepatitis C virus (HCV) prevalence; *δ* : proportion of HCV‐prevalent cases that are diagnosed; *T* : total population size; *b^D^* : bias parameter for the number of diagnosed people who inject drugs (PWID) recently; *b^δ^* : bias parameter for proportion diagnosed

All subgroups were modelled simultaneously. Estimation of unknown parameters of interest was based on the joint posterior distribution, with likelihood a product of independent binomial likelihoods for the NSP data and independent normal likelihoods for the stage 1 estimates (Supporting information, Appendix S4 and OpenBUGS model code in Supporting information, Appendix S6). Figure 3 presents schematically the relationship between the unknown parameters and the data sources.

Table 2 gives details of the prior distributions and constraints that were specified in the model.

**Table 2:** Prior assumptions for the parameters in the stage 2 model.

Parameter	Prior assumption	Comment
*ρ_R,d_*	Posterior distribution of recent PWID population size given by the CR study 16	
*π*_*R*,*d*_, *π*_*NR*,*d*_, *δ*_*R*,*d*_, *δ*_*NR*,*d*_, *ρ*_*NR*,*d*_	Uniform (0,1)	Flat prior distribution
$\text{log}\left(b_{a}^{D}\right),\;b_{a}^{\delta }$	Normal (0,10000) With a lower bound of log (0.5) and an upper bound of log 5	Flat prior distribution
		Values outside the range of 0.5–5 were thought to be implausible
	Also, log $\left(b_{1}^{D}\right)$ < log $\left(b_{2}^{D}\right)$, $b_{1}^{\delta }$ < $b_{2}^{\delta }$	
		It was expected that any bias would be greater in the older age‐group (*a* = 2) than the younger age‐group (*a* = 1)

CR = capture–recapture; people who inject drugs.

## Results

### Stage 1

#### Estimated number of HCV‐diagnosed recent and non‐recent PWID

The estimated numbers of HCV‐diagnosed recent and non‐recent PWID in Scotland in 2009 are 6639 (95% CI = 5205–8282) and 12 593 (95% CI = 10 859–14 513), respectively, totalling 19 268 (95% CI = 19 259–20 512) HCV‐diagnosed PWID (Table 3).

**Table 3:** Estimated number of total, people who inject drugs (PWID) recently and non‐recently among hepatitis C virus (HCV) diagnosed in Scotland during 2009 by region of residence, gender and age [estimated numbers are posterior medians and 95% credible intervals (CI)].

			Observed number diagnosed with HCV			Estimated number diagnosed with HCV			
			Total	Known PWID	Unknown risk	PWID (95% CI)	Recent PWID (95% CI)	Non‐recent PWID (95% CI)	Proportion of PWID who are recent PWID (95% CI)
Glasgow	Males	15–34 years	1350	993	307	1231 (1191, 1277)	459 (382, 545)	771 (682, 860)	0.37 (0.31, 0.44)
		35–64 years	4694	3156	1302	4113 (3929, 4336)	1303 (995, 1650)	2805 (2441, 3187)	0.32 (0.24, 0.40)
	Females	15–34 years	999	713	242	915 (891, 942)	298 (241, 360)	617 (423, 856)	0.33 (0.26, 0.39)
		35–64 years	2087	1261	655	1732 (1632, 1851)	469 (335, 624)	1259 (1095, 1440)	0.27 (0.19, 0.36)
	Total (excluding 63 with unknown gender)		9130	6123	2506	7990 (7655, 8402)	2531 (1976, 3154)	5452 (4796, 6146)	0.32 (0.25, 0.39)
Rest of Scotland	Males	15–34 years	2666	1742	840	2416 (2326, 2524)	1055 (901, 1227)	1359 (1178, 1547)	0.44 (0.37, 0.51)
		35–64 years	6324	3525	2438	5272 (4908, 5717)	1833 (1373, 2372)	3421 (2856, 4074)	0.35 (0.26, 0.44)
	Females	15–34 years	1689	1102	538	1542 (1486, 1606)	599 (499, 711)	941 (824, 1060)	0.39 (0.32, 0.46)
		35–64 years	2601	1244	1145	2048 (1867, 2268)	617 (423, 856)	1417 (1170, 1722)	0.30 (0.21, 0.41)
	Total (excluding 143 with unknown gender)		13 280	7613	4961	11 278 (10 600, 12 111)	4107 (3218, 5139)	7139 (6053, 8380)	0.36 (0.28, 0.45)
Total (excluding 206 with unknown gender)			22 410	13 736	7467	19 268 (18 259, 20 512)	6639 (5205, 8282)	12 593 (10 859, 14 513)	0.34 (0.27, 0.43)

Seventy‐three per cent (95% CI = 61–91%) of diagnosed individuals with unknown risk at diagnosis are estimated to be PWID (*p*_3_ in Fig. 1). This increases the total number of diagnosed PWID by approximately 40% compared with ignoring this unknown risk group, from 61% to 86% of all those diagnosed.

The estimated proportion of diagnosed PWID who are recent PWID varies from 0.27 to 0.44 across demographic groups. Lower proportions are estimated for the older age group compared with the younger, for women compared with men and for those living in Glasgow compared with the rest of Scotland.

### Stage 2

The results presented in the following sections are from the baseline model with bias parameters. Results from other models are given in the Sensitivity analysis section.

#### Estimated number of recent and non‐recent PWID

The estimated numbers of non‐recent PWID are considerably higher than of recent PWID, particularly in the older age group (Table 4). The number of recent PWID in Scotland in 2009 is 15 411 (95% CI = 13 243–17 134) compared to 67 246 (95% CI = 45 200–102 662) non‐recent PWID. Note (Fig. 2) that the posterior distributions for recent PWID are slightly bimodal, reflecting the bimodal CR prior.

**Table 4:** Posterior medians and 95% credible intervals for people who inject drugs (PWID) group size, PWID group size per 1000 population, hepatitis C virus (HCV) prevalence and number of HCV prevalent for recent and non‐recent PWID in Scotland during 2009, by region of residence, gender and age.

			PWID group size	PWID group size per 1000 population (ρ)	HCV prevalence % (π)	Total number of HCV prevalent (Tρπ)
Glasgow	Recent PWID	Males 15–34	1470 (1206, 1994)	8.5 (7.0, 11.6)	55% (49%, 61%)	816 (651, 1097)
		Males 35–64	1400 (1026, 1746)	6.1 (4.5, 7.6)	76% (70%, 81%)	1064 (780, 1334)
		Females 15–34	662 (527, 896)	4.0 (3.2, 5.4)	73% (63%, 81%)	482 (373, 651)
		Females 35–64	355 (260, 450)	1.4 (1.0, 1.8)	77% (64%, 87%)	271 (195, 355)
		Total number	3933 (3371, 4626)	4.8 (4.1, 5.6)	67% (63%, 71%)	2655 (2256, 3102)
	Non‐recent PWID	Males 15–34	3515 (1952, 7422)	20.4 (11.3, 43.0)	58% (48%, 67%)	2018 (1152, 4177)
		Males 35–64	12 442 (7797, 20 750)	54.2 (34.0, 90.4)	81% (74%, 87%)	10 050 (6328, 16 674)
		Females 15–34	2645 (1472, 5736)	15.9 (8.8, 34.5)	65% (54%, 75%)	1709 (982, 3686)
		Females 35–64	7477 (4076, 15 871)	29.9 (16.3, 63.4)	69% (55%, 81%)	5112 (2876, 10 529)
		Total number	26 853 (17 200, 43 254)	32.8 (21.0, 52.8)	72% (66%, 78%)	19 388 (12 467, 30 899)
Rest of Scotland	Recent PWID	Males 15–34	5845 (4365, 6830)	11.7 (8.7, 13.6)	37% (33%, 41%)	2157 (1618, 2592)
		Males 35–64	2225 (1915, 2965)	2.8 (2.4, 3.7)	53% (48%, 59%)	1199 (988, 1595)
		Females 15–34	2732 (2042, 3204)	5.6 (4.2, 6.5)	42% (36%, 48%)	1129 (848, 1385)
		Females 35–64	637 (479, 798)	0.7 (0.6, 0.9)	63% (52%, 74%)	397 (290, 524)
		Total number	11 463 (9564, 12 864)	4.3 (3.6, 4.8)	43% (40%, 46%)	4901 (4141, 5591)
	Non‐recent PWID	Males 15‐34	7698 (4367, 15 192)	15.4 (8.7, 30.3)	38% (31%, 45%)	2894 (1720, 5562)
		Males 35–64	19 420 (12 374, 32 856)	24.0 (15.3, 40.7)	50% (41%, 59%)	9722 (6460, 15 969)
		Females 15–34	5221 (2939, 11 186)	10.7 (6.0, 22.9)	41% (31%, 51%)	2130 (1280, 4373)
		Females 35–64	6552 (3838, 12 882)	7.6 (4.5, 15.0)	66% (51%, 79%)	4286 (2634, 8150)
		Total number	39 995 (27 028, 62 172)	15.1 (10.2, 23.4)	49% (43%, 55%)	19 526 (13 392, 29 931)
All Scotland	Recent PWID		15 411 (13 243, 17 134)	4.4 (3.8, 4.9)	49% (47%, 52%)	7559 (6579, 8501)
	Non‐recent PWID		67 246 (45 200, 102 662)	19.4 (13.0, 29.5)	58% (53%, 63%)	39 121 (26 310, 59 094)
	15–34 years		29 969 (22 499, 47 319)	22.5 (16.9, 35.6)	45% (41%, 48%)	13 449 (10 144, 21 347)
	35–64 years		51 655 (35 325, 78 190)	24.1 (16.5, 36.4)	64% (58%, 69%)	32 769 (22 516, 49 243)
	Males		55 019 (40 469, 78 958)	32.1 (23.6, 46.1)	55% (50%, 60%)	30 454 (22 184, 43 640)
	Females		27 157 (18 999, 42 156)	15.4 (10.8, 23.9)	59% (53%, 65%)	16 020 (11 188, 24 912)
	Glasgow		30 794 (21 126, 47 275)	37.6 (25.8, 57.7)	72% (66%, 76%)	22 045 (15 100, 33 615)
	Rest of Scotland		51 346 (38 310, 73 703)	19.3 (14.4, 27.7)	48% (43%, 52%)	24 400 (18 257, 34 892)
	Total		82 536 (60 408, 118 205)	23.8 (17.4, 34.0)	57% (52%, 61%)	46 657 (33 812, 66 803)

#### HCV prevalence estimates

Prevalence estimates vary between subgroups from 37 to 81%, with a higher prevalence in Glasgow than in the rest of Scotland, and in the older versus younger age groups (Table 4). In Glasgow, the prevalence is higher in non‐recent than recent PWID in men, but the reverse is found in women. Outside Glasgow, HCV prevalences for recent and non‐recent PWID are similar. The estimated total number of HCV‐prevalent cases in Scotland in 2009 is 46 657 (95% CI = 33 812–66 803), involving 7559 (95% CI = 6579–8501) recent and 39 121 (95% CI = 26 310–59 094) non‐recent PWID.

#### HCV diagnosed and undiagnosed estimates

The estimated proportion diagnosed ranges from 30 to 56% and is generally higher in the younger than the older age groups (Table 5).

**Table 5:** Posterior medians and 95% credible intervals for number of hepatitis C virus (HCV) prevalent diagnosed and undiagnosed cases for people who inject drugs (PWID) recently and non‐recently in Scotland during 2009, by region of residence, gender and age.

			Number of HCV diagnosed (Tρπδ)	Number of HCV undiagnosed (Tρπ(1 − δ))	Proportion diagnosed (δ)
Glasgow	Recent PWID	Males 15–34	356 (205, 547)	464 (283, 742)	43% (25%, 62%)
		Males 35–64	318 (212, 508)	731 (469, 1008)	30% (20%, 48%)
		Females 15–34	231 (133, 356)	250 (143, 417)	48% (28%, 67%)
		Females 35–64	113 (72, 180)	152 (84, 238)	43% (27%, 64%)
		Total number	1033 (695, 1446)	1611 (1162, 2128)	40% (26%, 54%)
	Non‐recent PWID	Males 15–34	873 (677, 1033)	1144 (432, 3181)	43% (24%, 63%)
		Males 35–64	3777 (3484, 4045)	6265 (2711, 12 802)	38% (23%, 57%)
		Females 15–34	683 (555, 785)	1028 (390, 2927)	40% (21%, 61%)
		Females 35–64	1610 (1471, 1745)	3499 (1313, 8887)	32% (16%, 55%)
		Total number	6937 (6446, 7381)	12 443 (5868, 23 697)	36% (23%, 53%)
Rest of Scotland	Recent PWID	Males 15–34	796 (458, 1204)	1319 (839, 1857)	38% (22%, 54%)
		Males 35–64	513 (357, 806)	684 (431, 1055)	43% (29%, 62%)
		Females 15–34	445 (255, 671)	666 (418, 968)	40% (23%, 58%)
		Females 35–64	156 (99, 251)	233 (132, 362)	40% (25%, 61%)
		Total number	1937 (1281, 2685)	2922 (2122, 3843)	40% (26%, 54%)
	Non‐recent PWID	Males 15–34	1614 (1193, 1975)	1278 (467, 3650)	56% (34%, 74%)
		Males 35–64	4725 (4191, 5232)	4991 (2008, 11 056)	49% (30%, 69%)
		Females 15–34	1094 (857, 1297)	1036 (363, 3127)	51% (28%, 72%)
		Females 35–64	1877 (1631, 2119)	2405 (850, 6212)	44% (24%, 68%)
		Total number	9301 (8379, 10 164)	10 215 (4719, 20 095)	48% (33%, 65%)
All Scotland	Recent PWID		2973 (1992, 4098)	4537 (3386, 5846)	40% (26%, 53%)
	Non‐recent PWID		16 237 (14 965, 17411)	22 872 (11 008, 42 050)	43% (30%, 59%)
	15–34 years		6095 (5951, 6238)	7354 (4056, 15 239)	46% (29%, 60%)
	35–64 years		13 121 (12 535, 13 710)	19 651 (9451, 36 045)	41% (27%, 59%)
	Males		13 000 (12 457, 13 538)	17 455 (9228, 30 603)	44% (31%, 59%)
	Females		6217 (5943, 6492)	9802 (5003, 18 692)	40% (26%, 56%)
	Glasgow		7973 (7701, 8245)	14 071 (7152, 25 614)	36% (24%, 53%)
	Rest of Scotland		11 244 (10 703, 11 782)	13 159 (7080, 23 591)	46% (33%, 61%)
	Total		19 216 (18 614, 19 823)	27 434 (14 636, 47 564)	42% (30%, 57%)

The estimated total number of undiagnosed‐HCV‐prevalent PWID in Scotland in 2009 is 27 434 (95% CI = 14 636–47 564), with more than 80% of undiagnosed cases being non‐recent PWID and more than 65% in the older age‐group.

#### Bias parameter estimates

Estimates of the number of diagnosed recent PWID generated from the DiagDat/TrtDat data in stage 1 are larger than expected, based on the other data sources, by a factor of 1.30 (95% CI = 0.87–2.23) in the younger age group and 3.81 (95% CI = 2.45–4.93) in the older age group. This bias parameter estimate is clearly larger in the older age group than the younger, suggesting there are fewer than a third as many diagnosed recent PWID aged > 35 than estimated in stage 1.

The estimated odds ratio of the NSP reported to the ‘true’ diagnosed proportion is 0.99 (95% CI = 0.53–2.11) in 15–34‐year‐olds and 1.75 (95% CI = 0.85–3.06) in 35–64‐year‐olds. Although there is a suggestion of an age difference in the bias parameter estimates for the NSP diagnosed data, due to uncertainty there is no clear evidence of a difference.

### Model fit

The overall model fit was assessed using deviance summaries. The baseline model provided a nearly exact fit, as the numbers of data points and parameters are similar (Table 6).

**Table 6:** Goodness‐of‐fit statistics for model parameters. $\hat{D}$ is the deviance evaluated at the maximum likelihood result, $\bar{D}$ is the posterior mean deviance, *p_D_* is the number of parameters and DIC is the deviance information criterion 23, 24.

	Number of data items	$\hat{D}$	$\bar{D}$	*p*_*D*_	DIC
Baseline model	48	2.43	47	44	91
NSP (HCV prevalence)	16	0.37	16	15	31
NSP (proportion diagnosed)	16	1.01	15	14	30
DiagDat/TrtDat	16	1.04	16	15	30

A model that fits well $\bar{D}$ will be approximately equal to the number of data items and $\hat{D}$ will be approximately equal to the number of degrees of freedom (the difference between the number of data items and the number of parameters). The DIC is equal to the posterior mean deviance $\bar{D}$ with the addition of a penalty term for the number of parameters *p_D_*. DiagDat = Hepatitis C Diagnosis Database; TrtDat = Drugs Misuse Database; HCV = hepatitis C virus; NSP = Needle Exchange Surveillance Initiative.

### Sensitivity analysis

The inclusion of bias‐adjustment parameters was driven by expert knowledge of the data sources and their potential biases. No direct empirical evidence was available to inform the bias parameters; hence, the expert knowledge comprised plausible upper and lower bounds for their prior distributions (Table 2). To assess sensitivity to this expert judgement, alternative models including one in which the data sources were assumed to be unbiased, were explored:

Sensitivity 1: Model with unbounded bias parameters;
Sensitivity 2: Model without bias parameters; and
Sensitivity 3: Model without either bias parameters or CR informative prior for *ρ*_*R*._

When the bounds for the bias parameters are removed the non‐recent PWID estimates increase greatly, due to a higher upper limit for the 95% credible interval estimate of the risk group size (sensitivity 1).

There is evidence that without bias adjustment (sensitivity 2) there is some lack of fit, with estimates of the number of recent PWID from this model being in conflict with those from the CR prior (17 811 and 15 618, respectively) (Table 7 and Fig. 2). Without the CR prior (sensitivity 3), the estimated number of recent PWID are even higher (27 977), further supporting the hypothesis that the lack of fit was due largely to this conflict between the CR study and the other data.

**Table 7:** Posterior medians and 95% credible intervals for people who inject drugs (PWID) group size, number of hepatitis C virus (HCV) prevalent and number of HCV undiagnosed from sensitivity analyses.

	PWID group size		Total number of HCV prevalent		Number of HCV undiagnosed	
	Recent PWID	Non‐recent PWID	Recent PWID	Non‐recent PWID	Recent PWID	Non‐recent PWID
Baseline model	15 411 (13 243, 17 134)	67 246 (45 200, 102 662)	7559 (6579, 8501)	39 121 (26 310, 59 094)	4537 (3386, 5846)	22 872 (11 008, 42 050)
Sensitivity 1	15 367 (13 213, 17 087)	79 874 (44 571, 234 209)	7530 (6557, 8469)	46 940 (26 183,140 769)	4808 (3190, 6440)	30 373 (11 079, 122 917)
Sensitivity 2	17 811 (16 434, 19 357)	43 264 (37 890, 49 866)	9123 (8355, 10 001)	24 459 (21 842, 27 666)	4725 (4205, 5299)	11 862 (9616, 14 768)
Sensitivity 3	27 977 (24 445, 31 969)	43 241 (37 871, 49 819)	14 723 (12 933, 16 679)	24 460 (21 837, 27 668)	8078 (6805, 9588)	11 864 (9611, 14 776)

The main results and conclusions presented were based on the baseline model with bias adjustment parameters, as this gave the best model fit according to deviance statistics when incorporating all available relevant sources of information (Supporting information, Appendix S5).

## Discussion

### Key findings

For the first time, using MPES, we have obtained estimates of the size of the HCV undiagnosed populations, which are particularly valuable for the planning of future health‐service demands and for identifying specific subgroups to target in prevention programmes. Other modelling has demonstrated that new HCV treatments could have a substantial impact on reducing HCV transmission among PWID 6, 25. Accurate assessment of the magnitude of that effect, as well as implementation of treatment strategies, will require reliable knowledge of the diagnosed and undiagnosed PWID populations. We estimated that of the 46 000 prevalent HCV infections among PWID in Scotland in 2009, 59% were undiagnosed and 83% (95% CI = 75–89%) of the undiagnosed had not injected that year. While some non‐recent PWID will be in contact with drug treatment services, an appreciable number may not. Reaching this population may prove challenging, but it is necessary to implement diagnosis and treatment programmes. Our analysis has also highlighted a need to target diagnosis programmes towards older age groups. We estimated that 71% (95% CI = 58–85%) of undiagnosed PWID in 2009 were aged 35–64 years, compared with 55% of all new HCV diagnoses in Scotland in 2009–12 in the same age group 26. Furthermore, as these older individuals are at greater risk of progressing to advanced stages of HCV disease, they have a pressing need for prompt treatment.

In Glasgow, HCV prevalence estimates are especially high in the older group due to a historical injecting epidemic which started in the early 1980s, and resulted in a rapid rise in the number of PWID before the establishment of needle/syringe exchange in the city.

Linkage of TrtDat to DiagDat enabled a better‐informed estimate of the number of PWID among the HCV‐diagnosed for use in the evidence synthesis than was obtainable from DiagDat alone. Through modelling the probability of linkage to TrtDat and the recent/non‐recent status of PWID explicitly, estimates of the size of subgroups that were not observed directly (e.g. PWID in the unknown risk group) were obtained. This increased the estimated number of diagnosed PWID from 61 to 86% of all those diagnosed, comparable to a similar estimate from a CR study of the Scottish HCV‐diagnosed population 22.

### Limitations

Producing reliable estimates of the number of individuals with anti‐HCV antibodies depends upon information on the size of the PWID population. The CR study provides estimates of the number of recent PWID, but no data on the size of the non‐recent PWID population exist. This is, by nature, a difficult risk group to identify and survey. Recent PWID were estimated to account for only 19% (95% CI = 13–26%) of the ever‐PWID population, similar to modelling projections for Scotland for 2010 of 19% 25 but smaller than estimates for England in 2005 of 40% 12.

The definition of non‐recent PWID is variable, even among the data sources used here, and cannot be interpreted as long‐term cessation of injecting. The CR study provides estimates of the number of PWID injecting during a particular year (2009), but in other data sources ‘recent’ was defined as injecting in the last month. However, this definition of recent does not capture all infrequent but at risk of continuing injectors, and nor does the CR definition of ‘last year’ injectors, due to the high uptake of methadone treatment in the PWID population. We were limited by the definitions in the data available; however, a challenge for the future is the collection of data in which a broader definition of recent PWID, which includes infrequent injectors and reflects that people temporarily cease injecting due to opioid substitution therapy and prison, is used.

The sensitivity analyses highlighted a discrepancy between the number of recent PWID estimated by the CR study alone and the number suggested by the other data, which was resolved by inclusion of bias‐adjustment parameters. The propensity of a recent PWID to contact drug treatment services and hence be reported in TrtDat may not be the only reason for bias; the regression analysis may not have captured fully the characteristics that distinguish recent from non‐recent PWID. In the absence of data on timings or characteristics of the transition from injecting to non‐injecting, a more comprehensive modelling of injecting careers, allowing prediction of the recent/non‐recent status at any point in time, was impossible. Furthermore, to discriminate more clearly between the sensitivity analyses and estimate more accurately the magnitude of the biases in the data would require improved external data, ideally on the sizes of the recent and non‐recent PWID populations, which are currently non‐existent due to the challenge of surveying these populations.

### Findings in relation to other evidence

We have presented estimates of HCV‐antibody prevalence in PWID in Scotland from the combined use of survey and surveillance data relating to PWID and HCV prevalence. The flexibility of the MPES approach allowed us to combine the information from each data source simultaneously; to account for any potential bias; and to propagate the full uncertainty of each contributing data item through to the final estimates. This approach overcomes the limitations of more traditional methods of prevalence estimation 13, 27. MPES methods have been employed successfully to estimate the prevalence and incidence of other diseases, including HIV 9, toxoplasmosis 8 and influenza 28, 29, as well as for HCV prevalence estimation in other countries 12, 30, 31. To our knowledge, however, this is the first synthesis that allows estimation of undiagnosed HCV prevalence. Evidence synthesis that accounts for expert knowledge of biases and other limitations of available data may be of value to other countries, particularly those with a mixed evidence base for HCV infection.

### Implications

HCV testing in drug treatment services has been found recently to be effective in increasing the numbers of PWID diagnosed in Scotland 32, 33. Targeting older individuals with a history of injecting drug use through primary care can also be an effective case‐finding approach 34. However, such approaches will require fully engaged general practitioners (GPs) and community‐setting practitioners in high HCV‐prevalence areas, and widespread adoption, to diagnose the vast majority of PWID.

Our modelling has focused upon HCV in the PWID population. While these individuals account for the majority of the HCV burden, the contribution of other risk groups may also be important. HCV prevalence varies by ethnicity and it is thought that South Asian individuals may have an increased prevalence 12. Future work will extend the evidence synthesis to include ethnicity, thus estimating the prevalence of undiagnosed HCV for the whole population in Scotland.

## Declaration of interests

None.

## Supporting information



Appendix S1 Logistic regression for estimating the probability that an HCV‐diagnosed “ever PWID” in TrtDat in 1995‐2008 is a recent PWID in 2009.

Appendix S2 Data.

Appendix S3 Bias adjustment parameters in MPES model.

Appendix S4 Relationship between data and model parameters in Stage 2 MPES model.

Appendix S5 Sensitivity analyses.

Appendix S6 OpenBUGS model code for Stage 1 and Stage 2.
