# Supplementary material for: Estimating the number of people with hepatitis C virus who have ever injected drugs and have yet to be diagnosed: an evidence synthesis approach for Scotland
Source: Addiction. 2015 Jun 8;110(8):1287–300. doi: 10.1111/add.12948 (PMC4744705; doi:10.1111/add.12948)
Supplement: Supplementary file 1 — Appendix S1 Logistic regression for estimating the probability that an HCV‐diagnosed “ever PWID” in TrtDat in 1995‐2008 is a recent PWID in 2009. [file ADD-110-1287-s001.doc]

**Appendix 1 - Logistic** **regression for estimating the probability that an HCV-diagnosed “ever PWID” in SDMD in 1995-2008 is a recent PWID in 2009.**

Using data on all HCV-diagnosed “ever PWID” in SDMD from 1995 to 2009 who were alive mid-2009, the probability of being a recent PWID, *q* can be expressed in terms of the covariates age (A), gender (G), health board (GGC or Rest of Scotland) (B), years since starting injecting (T), years since HCV diagnosis (D) and year of last SDMD record (Y) as follows :

$$\text{logit}(q_{k})=\alpha+\beta_{\text{A}}\text{A}_{k}+\beta_{\text{G}}\text{G}_{k}+\beta_{\text{B}}\text{B}_{k}+\beta_{T}T_{k}+\beta_{\text{D}}\text{D}_{k}+\beta_{\text{Y}}\text{Y}_{k}$$

The resulting coefficient estimates ($\hat{\alpha},\hat{\beta}_{\text{A}},\hat{\beta}_{\text{G}},\hat{\beta}_{\text{B}},\hat{\beta}_{T},\hat{\beta}_{\text{D}}$) are used to calculate the probability that each individual *l* “ever PWID” in SDMD from 1995 to 2008 is a recent PWID in 2009 ($c_{l}$),

$$\text{logit}(c_{l})=\hat{\alpha}+\hat{\beta}_{\text{A}}\text{A}'_{l}+\hat{\beta}_{\text{G}}\text{G}_{l}+\hat{\beta}_{\text{B}}\text{B}_{l}+\hat{\beta}_{T}\text{T}'_{l}+\hat{\beta}_{\text{D}}\text{D}'_{l}$$

where $\text{A}'\text{, }\text{T}'\text{, }\text{D}'$ are the covariates age, years since starting injecting and years since HCV diagnosis respectively updated to the values they would be in 2009. The total number of recent PWID in this group of ever PWID in SDMD in 1995 to 2008 is given by$\sum_{l} I_{l}$, where *I_l_* is Bernoulli distributed: *I _l_*~ Bern(*c_l_*).The probability that an ever PWID in SDMD in 1995 to 2008 with a known PWID risk on SHCDD is a recent PWID in 2009, *p_12_* (Figure 1), is estimated by $\sum_{l=1}^{E_{IDU}} I_{l}/E_{IDU}$ where $E_{IDU}=$the number of diagnosed with known PWID risk at diagnosis who are ever PWID in SDMD 1995 to 2008. Similarly the probability that an ever PWID in SDMD in 1995 to 2008 with an unknown PWID risk at diagnosis is a recent PWID in 2009, *p_17_*, is estimated by $\sum_{l=1}^{E_{NK}} I_{l}/E_{NK}$ where $E_{NK}=$the number of diagnosed with unknown PWID risk at diagnosis who are ever PWID in SDMD 1995 to 2008. Estimates of the regression coefficients are given in the table below.

**Table A1.1**: Posterior medians and 95% credible intervals for logistic regression coefficients for the probability that an “ever PWID” in SDMD in 1995-2009 is a recent PWID

|  | **n** | **OR**  **(Posterior median)** | **95% CI** | |
| --- | --- | --- | --- | --- |
| **Constant** |  | 3.08 | (2.46, | 3.86) |
| **Age** |  |  |  |  |
| 15-24 | 1347 | 1.00 |  |  |
| 25-29 | 2460 | 0.73 | (0.63, | 0.85) |
| 30-34 | 2751 | 0.66 | (0.57, | 0.77) |
| 35-39 | 1978 | 0.64 | (0.54, | 0.76) |
| 40+ | 1625 | 0.61 | (0.50, | 0.74) |
| **Year of last SDMD registration** |  |  |  |  |
| 1995-1999 | 1056 | 1.23 | (1.02, | 1.48) |
| 2000-2004 | 3329 | 1.02 | (0.88, | 1.18) |
| 2005-2008 | 4663 | 0.79 | (0.69, | 0.91) |
| 2009 | 1113 | 1.00 |  |  |
| **Gender** |  |  |  |  |
| Female | 3230 | 1.00 |  |  |
| Male | 6931 | 1.47 | (1.34, | 1.60) |
| **Health Board** |  |  |  |  |
| Rest of Scotland | 5672 | 1.00 |  |  |
| GGC | 4489 | 0.69 | (0.63, | 0.75) |
| **Years since 1^st^ injected** |  |  |  |  |
| 0-2 | 896 | 1.00 |  |  |
| 3-5 | 1552 | 0.55 | (0.46, | 0.66) |
| 6-10 | 3097 | 0.47 | (0.40, | 0.56) |
| 11-15 | 2211 | 0.43 | (0.36, | 0.52) |
| 16-20 | 1302 | 0.35 | (0.29, | 0.44) |
| 21+ | 1103 | 0.31 | (0.24, | 0.39) |
| **Years since HCV diagnosis** |  |  |  |  |
| After SDMD | 2784 | 1.00 |  |  |
| 0 | 1139 | 0.88 | (0.76, | 1.02) |
| 1+ years | 6238 | 0.84 | (0.76, | 0.93) |
