# Supplementary material for: Estimating the number of people with hepatitis C virus who have ever injected drugs and have yet to be diagnosed: an evidence synthesis approach for Scotland
Source: Addiction. 2015 Jun 8;110(8):1287–300. doi: 10.1111/add.12948 (PMC4744705; doi:10.1111/add.12948)
Supplement: Supplementary file 2 — Appendix S2 Data. [file ADD-110-1287-s002.doc]

**Appendix 3 - Bias adjustment parameters in MPES model.**

Bias parameter for Stage 1 estimates of number of HCV diagnosed

To allow for the potential overestimate in the number of diagnosed recent PWID estimated in Stage 1 (due to the likely bias in SDMD towards recent rather than non-recent PWID), whilst at the same time assuming no bias in the total number of diagnosed PWID, a bias-adjustment parameter was included in the model.

The estimated number of diagnosed recent PWID,$y_{R,d}^{D}$, obtained from Stage 1, was taken as a realization from a Normal distribution with mean $\mu_{R,d}^{D}$ and known variance$\sigma_{R,d}^{2}$, equal to the posterior variance of$y_{R,d}^{D}$. The mean,$\mu_{R,d}^{D}$, was expressed in terms of the ‘true’ number of diagnosed recent PWID, $T_{d}\rho_{R,d}\pi_{R,d}\delta_{R,d}$and an age-specific bias parameter, $b_{a}^{D}$ (*a*=1,2), such that,

$\text{log}(\mu_{R,d}^{D})=\text{log}(T\rho_{R,d}\pi_{R,d}\delta_{R,d})+\log(b_{a}^{D})$.

$b_{a}^{D}$ represents the ratio of the SDMD-estimated number of diagnosed recent PWID to the ‘true’ number of diagnosed recent PWID.

Bias parameter for diagnosed proportion from NESI

To account for the potential bias due to NESI participants being more likely to have been tested for HCV then non-NESI PWID, the diagnosed proportion estimated from NESI,$\delta_{i,d}^{*}$, was expressed in terms of the ‘true’ unbiased diagnosed proportion,$\delta_{i,d}$ and an age-specific bias parameter $b_{a}^{\delta}(a=1,2)$:

$\text{logit}(\delta_{i,d}^{*})=\text{logit}(\delta_{i,d})+b_{a}^{\delta}$.

$b_{a}^{\delta}$ represents the log odds ratio of the NESI-estimated diagnosed proportion relative to the ‘true’ diagnosed proportion.

The bias was assumed to be equal for recent and non-recent PWID, due to a lack of identifiability arising in the non-recent PWID parameter estimates if differing biases were assumed. The recent PWID parameters are identifiable however, due to the additional contribution of information from the CRC estimates.
