# Supplementary material for: Estimating the number of people with hepatitis C virus who have ever injected drugs and have yet to be diagnosed: an evidence synthesis approach for Scotland
Source: Addiction. 2015 Jun 8;110(8):1287–300. doi: 10.1111/add.12948 (PMC4744705; doi:10.1111/add.12948)
Supplement: Supplementary file 3 — Appendix S3 Bias adjustment parameters in MPES model. [file ADD-110-1287-s003.doc]

**Appendix 2 – Data**

**Table A2.1:** Estimated number of current PWID in Scotland 2009. Posterior median estimates (and 95% credible intervals) from capture-recapture study (15)

|  | Males | | Females | |  |
| --- | --- | --- | --- | --- | --- |
|  | <35 years | 35+ years | <35 years | 35+ years | Total |
| GGC | 1694 (1217, 2179) | 1300 (935, 1697) | 746 (529, 974) | 334 (244, 441) | 4116  (3004, 5145) |
| Rest of  Scotland | 5561 (4138, 6822) | 2614 (1962, 3278) | 2564 (1923, 3157) | 631 (471, 810) | 11450  (8641, 13794) |
| Total | 7284 (5391, 8931) | 3929 (2924, 4935) | 3323 (2471, 4097) | 968 (724, 1238) | 15618  (11715, 18809) |

**Table A2.2**: Data from NESI survey 2008/2009 (16).

|  | Number  surveyed and tested for HCV antibodies | Number  tested HCV antibody positive | Observed HCV prevalence | Number tested HCV antibody positive, who also reported had previous positive test | Observed proportion HCV diagnosed |
| --- | --- | --- | --- | --- | --- |
| **Recent PWID** |  |  |  |  |  |
| **GGC**  Male 15-34 yrs | 220 | 122 | 55% | 54 | 44% |
| 35-64 yrs | 217 | 165 | 76% | 69 | 42% |
| Female 15-34 yrs | 94 | 69 | 73% | 34 | 49% |
| 35-64 yrs | 43 | 33 | 77% | 17 | 52% |
| Total | 574 | 389 | 68% | 174 | 45% |
| **Rest of Scotland**  Male 15-34 yrs | 554 | 205 | 37% | 76 | 37% |
| 35-64 yrs | 299 | 161 | 54% | 93 | 58% |
| Female 15-34 yrs | 248 | 102 | 41% | 39 | 38% |
| 35-64 yrs | 63 | 40 | 63% | 21 | 53% |
| Total | 1164 | 508 | 44% | 229 | 45% |
| **Non-recent PWID** |  |  |  |  |  |
| **GGC**  Male 15-34 yrs | 98 | 57 | 58% | 25 | 44% |
| 35-64 yrs | 134 | 109 | 81% | 56 | 51% |
| Female 15-34 yrs | 79 | 52 | 66% | 21 | 40% |
| 35-64 yrs | 44 | 31 | 70% | 14 | 45% |
| Total | 355 | 249 | 70% | 116 | 47% |
| **Rest of Scotland**  Male 15-34 yrs | 168 | 64 | 38% | 36 | 56% |
| 35-64 yrs | 116 | 59 | 51% | 37 | 63% |
| Female 15-34 yrs | 91 | 38 | 42% | 20 | 53% |
| 35-64 yrs | 43 | 29 | 67% | 17 | 59% |
| Total | 418 | 190 | 45% | 110 | 58% |
