# Supplementary material for: Estimating the number of people with hepatitis C virus who have ever injected drugs and have yet to be diagnosed: an evidence synthesis approach for Scotland
Source: Addiction. 2015 Jun 8;110(8):1287–300. doi: 10.1111/add.12948 (PMC4744705; doi:10.1111/add.12948)
Supplement: Supplementary file 4 — Appendix S4 Relationship between data and model parameters in Stage 2 MPES model. [file ADD-110-1287-s004.doc]

**Appendix 4 - Relationship between data and model parameters in Stage 2 MPES model**.

| Data Sources | No. of data points | Quantity of interest | Parameter form |
| --- | --- | --- | --- |
| NESI – HCV Prevalence | 8 | *π_R,d_* | $y_{R,d}^{\pi}\sim\text{Bin(}n_{R,d}^{\pi},\pi_{R,d})$ |
|  | 8 | *π_NR,d_* | $y_{NR,d}^{\pi}\sim\text{Bin(}n_{NR,d}^{\pi},\pi_{NR,d})$ |
| NESI – Proportion Diagnosed | 8 | *δ_R,d_* | $y_{R,d}^{\delta}\sim\text{Bin(y}_{R,d}^{\pi},\delta_{R,d}^{*})$ $\text{logit}(\delta_{R,d}^{*})=\text{logit}(\delta_{R,d})+b_{a}^{\delta}$ |
|  | 8 | *δ_NR,d_* | $y_{NR,d}^{\delta}\sim\text{Bin(y}_{NR,d}^{\pi},\delta_{NR,d}^{*})$ $\text{logit}(\delta_{NR,d}^{*})=\text{logit}(\delta_{NR,d})+b_{a}^{\delta}$ |
| SHCDD and linked SDMD – HCV diagnosed recent PWID | 8 | *ρ_R,d_, π_R,d_, δ_R,d_* | $y_{R,d}^{D}\sim\text{N(}\mu_{R,d}^{D},\sigma_{R,d}^{2})$ $\text{log}(\mu_{R,d}^{D})=\text{log}(T_{d}\rho_{R,d}\pi_{R,d}\delta_{R,d})+\log(b_{a}^{D})$ |
| SHCDD and linked SDMD – HCV diagnosed PWID | 8 | *ρ_R,d_, π_R,d_, δ_R,d_, ρ_N,d_, π_NR,d_, δ_NR,d_* | $y_{.d}^{D}\sim\text{N(}T_{d}\rho_{R,d}\pi_{R,d}\delta_{R,d}+T_{d}\rho_{NR,d}\pi_{NR,d}\delta_{NR,d},\sigma_{.d}^{2})$ |
