# Supplementary material for: Estimating the number of people with hepatitis C virus who have ever injected drugs and have yet to be diagnosed: an evidence synthesis approach for Scotland
Source: Addiction. 2015 Jun 8;110(8):1287–300. doi: 10.1111/add.12948 (PMC4744705; doi:10.1111/add.12948)
Supplement: Supplementary file 5 — Appendix S5 Sensitivity analyses. [file ADD-110-1287-s005.doc]

**Appendix 5 – Sensitivity Analyses**

Three sensitivity analyses were considered:

Sensitivity 1 – Model with unbounded bias parameters;

Sensitivity 2 – Model with bias parameters omitted;

Sensitivity 3 – Model without CRC informative prior for *ρ_R_*.

**Table A5.1**: Posterior medians and 95% credible intervals for PWID group size, number of HCV prevalent and number of HCV undiagnosed from sensitivity analyses.

|  | **PWID group size** | | **Total number of HCV prevalent** | | **Number of HCV undiagnosed** | |
| --- | --- | --- | --- | --- | --- | --- |
|  | Recent PWID | Non-recent PWID | Recent PWID | Non-recent PWID | Recent PWID | Non-recent PWID |
| Main analysis | 15411  (13243, 17134) | 67246  (45200, 102662) | 7559  (6579, 8501) | 39121  (26310, 59094) | 4537  (3386, 5846) | 22872  (11008, 42050) |
| Sensitivity1 | 15367  (13213, 17087) | 79874  (44571, 234209) | 7530  (6557,8469) | 46940  (26183,140769) | 4808  (3190,6440) | 30373  (11079,122917) |
| Sensitivity  2 | 17811  (16434, 19357) | 43264  (37890, 49866) | 9123  (8355, 10001) | 24459  (21842, 27666) | 4725  (4205, 5299) | 11862  (9616, 14768) |
| Sensitivity  3 | 27977  (24445, 31969) | 43241  (37871, 49819) | 14723  (12933, 16679) | 24460  (21837, 27668) | 8078  (6805, 9588) | 11864  (9611, 14776) |

**Table A5.2**: Goodness-of-fit statistics for the main analysis and the sensitivity analyses.

|  | Number of data items | $\widehat{D}$ | $\overline{D}$ | $p_{D}$ | DIC |
| --- | --- | --- | --- | --- | --- |
| Main analysis | 48 | 2.43 | 47 | 44 | 91 |
| Sensitivity 1 | 48 | 2.48 | 47 | 44 | 91 |
| Sensitivity 2 | 48 | 56.3 | 97 | 40 | 137 |
| Sensitivity 3 | 48 | 1.96 | 48 | 46 | 94 |

For a model that fits well $\overline{D}$ will be approximately equal to the number of data items and $\widehat{D}$ will be approximately equal to the number of degrees of freedom (the difference between the number of data items and the number of parameters). The DIC is equal to the posterior mean deviance $\overline{D}$ with the addition of a penalty term for the number of parameters$p_{D}$.

Sensitivity 1

When the bounds for the bias parameters are removed the uncertainty of the non-recent PWID estimates increase greatly, due to the upper limit of the 95% credible intervals for the risk group size becoming much larger. This is reflected in the estimates for the number of HCV prevalent and the number of undiagnosed (Table A5.1).

Sensitivity 2

When the bias-adjustment parameters are not included in the model there is evidence of a lack of fit, particularly for the SHCDD and linked SDMD data and to a lesser extent the NESI proportion diagnosed data (Table A5.3).

**Table A5.3**: Goodness-of-fit statistics for model parameters in sensitivity analysis 2.

|  | Number of data items | $\widehat{D}$ | $\overline{D}$ | $p_{D}$ | DIC |
| --- | --- | --- | --- | --- | --- |
| **Model (without bias adjustment)** | 48 | 56.3 | 97 | 40 | 137 |
| NESI (HCV prevalence) | 16 | 2.0 | 17 | 16 | 33 |
| NESI (proportion diagnosed) | 16 | 6.0 | 21 | 15 | 35 |
| SHCDD/SDMD | 16 | 48.0 | 59 | 11 | 70 |

For a model that fits well $\overline{D}$ will be approximately equal to the number of data items and $\widehat{D}$ will be approximately equal to the number of degrees of freedom (the difference between the number of data items and the number of parameters). The DIC is equal to the posterior mean deviance $\overline{D}$ with the addition of a penalty term for the number of parameters$p_{D}$.

The estimated size of the non-recent PWID population decreases and its 95% credible interval is reduced (Table A5.1). This is also seen in the estimates of prevalent cases and numbers undiagnosed.

When comparing the CRC prior with the posterior estimates for the recent PWID population size, there is also some evidence of a conflict between the data from the CRC study and the SHCDD and linked SDMD data which both contribute information to the estimation of size of the recent PWID risk group (Figure 3). For the older age-groups in GGC and for all the risk groups in the rest of Scotland, the posterior estimates are larger than the CRC prior.

Sensitivity 3

To explore further the conflict between the CRC estimates and the other data a sensitivity analysis was carried out, in which the informative prior for *ρ_R_* based on the CRC estimates was replaced with a vague prior, in the model with no bias parameters.

In all the subgroups, except the 15-34 years old from GGC, the estimated number of recent PWID is larger than when the CRC prior is used. Using a vague prior has allowed the resulting estimates for *ρ_R_*  to be such that the estimated number of diagnosed recent PWID ($T\rho_{R}\pi_{R}\delta_{R}$) are consistent with the SHCDD and linked SDMD data estimates. This leads to an increase in the estimate of the number of HCV prevalent cases in the recent PWID group.
