# Supplementary material for: Estimating the number of people with hepatitis C virus who have ever injected drugs and have yet to be diagnosed: an evidence synthesis approach for Scotland
Source: Addiction. 2015 Jun 8;110(8):1287–300. doi: 10.1111/add.12948 (PMC4744705; doi:10.1111/add.12948)
Supplement: Supplementary file 6 — Appendix S6 OpenBUGS model code for Stage 1 and Stage 2. [file ADD-110-1287-s006.doc]

**Appendix 6 – OpenBUGS model code for Stage 1 and Stage 2**

## Stage 1: Modelling of HCV diagnosis data (SHCDD, linked to SDMD) by region, gender,

## age to obtain estimates of number of HCV diagnosed recent & non-recent PWID

## RGA (Region/Gender/Age) / j

## 1: Glasgow, male, <35

## 2: Glasgow, male, 35+

## 3: Glasgow, female, <35

## 4: Glasgow, female, 35+

## 5: Elsewhere, male, <35

## 6: Elsewhere, male, 35+

## 7: Elsewhere, female, <35

## 8: Elsewhere, female, 35+

model

{

# -------------------------------------------------------------------------------------------------------------------------------------

# SHCDD data linked with SDMD, broken down into known/unknown risk group, PWID/non-PWID,

# SDMD/not SDMD, SDMD 1995-2008/SDMD 2009, ever/never injected, recent/non-recent, y.diag

# -------------------------------------------------------------------------------------------------------------------------------------

for(j in 1:8){

y.diag[1,1,j] ~ dpois(tot.diag[j])

tot.diag[j] <- sum(y.diag[13:22,1,j])+ sum(y.diag[13:22,2,j])+ y.diag[3,2,j] + y.diag[4,2,j]

for(i in 1:11){

y.diag[i+1,1,j] ~ dbin(prob[p[i]], y.diag[parent.i[i],parent.j[i],j])

y.diag[i+1,2,j] <- y.diag[parent.i[i],parent.j[i],j]-y.diag[i+1,1,j]

}

#------------------------------------------------------------------

# y.diag[13,,] estimated from regression below

#-------------------------------------------------------------------

for(i in 13:14) {

y.diag[i+1,1,j] ~ dbin(prob[p[i]], y.diag[parent.i[i],parent.j[i],j])

y.diag[i+1,2,j] <- y.diag[parent.i[i],parent.j[i],j]-y.diag[i+1,1,j]

}

}

#--------------------------------------------------------------------------------------------------------------------

# Zero count for y.diag[10,2] when RGA=3 so set y.diag[16,1]=0 for RGA=3

#--------------------------------------------------------------------------------------------------------------------

for(j in 1:2){

y.diag[16,1,j] ~ dbin(prob[p[15]], y.diag[parent.i[15],parent.j[15],j])

y.diag[16,2,j] <- y.diag[parent.i[15],parent.j[15],j]-y.diag[16,1,j]

}

for(j in 4:8){

y.diag[16,1,j] ~ dbin(prob[p[15]], y.diag[parent.i[15],parent.j[15],j])

y.diag[16,2,j] <- y.diag[parent.i[15],parent.j[15],j]-y.diag[16,1,j]

}

y.diag[16,1,3] <- 0

y.diag[16,2,3] <- 0

for(j in 1:8){

y.diag[17,1,j] ~ dbin(prob[p[16]], y.diag[parent.i[16],parent.j[16],j])

y.diag[17,2,j] <- y.diag[parent.i[16],parent.j[16],j]-y.diag[17,1,j]

#------------------------------------------------------------------

# y.diag[18,,] estimated from regression below

#-------------------------------------------------------------------

for(i in 18:21) {

y.diag[i+1,1,j] ~ dbin(prob[p[i]], y.diag[parent.i[i],parent.j[i],j])

y.diag[i+1,2,j] <- y.diag[parent.i[i],parent.j[i],j]-y.diag[i+1,1,j]

}

}

# --------------------------------------------------------------------------------------------------------------------------------

# Regression model for recent/non-recent status using ever injectors in SDMD 1995-2009

# Covariates age (15-24, 25-29, 30-34,35-39,40+), year of SDMD (1995-1999,2000-2004,

# 2005-2008,2009), gender (female/male), health board (elsewhere/Glasgow), year since 1st

# injected (0-2, 3-5, 6-10, 11-15, 16-20,21+), years since HCV diagnosis (After SDMD, 0,

# 1+ years)

# ---------------------------------------------------------------------------------------------------------------------------------

for(k in 1:899){

injectpm[k] ~ dbin(q[k],num[k])

logit(q[k]) <- alpha + beta.age[age[k]] + (beta.gender * gender[k])

+ (beta.glasgow * glasgow[k]) + beta.inj[inj[k]]

+ beta.diag[diag[k]] + beta.year[year[k]]

}

# -------------------------------------------------------------------------------------------------------------------------------------

# Use regression model to predict recent/non-recent status in 2009 for ever injector in SDMD

# 1995-2008, (age, years since 1st injecting & years since HCV diagnosis updated to 2009)

# -------------------------------------------------------------------------------------------------------------------------------------

for(l in 1:9048){

logit.c[l] <- alpha + beta.age[age2009[l]] + (beta.gender * gender2009[l])

+ (beta.glasgow * glasgow2009[l]) + beta.inj[inj2009[l]]

+ beta.diag[diag2009[l]] # (2009=beta.year[4]=0)

c[l] <- exp(logit.c[l] )/(1+exp(logit.c[l] ))

recent[l] ~ dbern(c[l])

for(j in 1:8){

recent.IDU[l,j] <- equals(RGA2009[l],j)*IDU[l]*recent[l]

recent.NK[l,j] <- equals(RGA2009[l],j)*(1-IDU[l])*recent[l]

}

}

# -------------------------------------------------------------------------------------------------------------------------------------

# Predictions for those with missing age at first injecting (assume time since first injecting equals

# mean time since first injecting)

#-------------------------------------------------------------------------------------------------------------------------------------

for(l in 1:357){

logit.c[l+9048] <- alpha + beta.age[age2009.m1[l]] + (beta.gender*gender.m1[l])

+ (beta.glasgow * glasgow.m1[l]) + beta.inj[3] + beta.diag[diag2009.m1[l]]

c[l+9048] <- exp(logit.c[l+9048])/(1+exp(logit.c[l+9048]))

recent[l+9048] ~ dbern(c[l+9048])

for(j in 1:8) {

recent.IDU[l+9048,j] <- equals(RGA2009.m1[l],j)*IDU.m1[l]*recent[l+9048]

recent.NK[l+9048,j] <- equals(RGA2009.m1[l],j)*(1-IDU.m1[l])*recent[l+9048]

}

}

#--------------------------------------------------------------------------------------------------------

# Estimated total recent & non-recent in 2009 (by known/unknown PWID)

#---------------------------------------------------------------------------------------------------------

for(j in 1:8){

tot.recent.IDU[j] <- sum(recent.IDU[,j])

tot.recent.NK[j] <- sum(recent.NK[,j])

y.diag[13,1,j] <- cut(tot.recent.IDU[j])

y.diag[13,2,j] <- y.diag[9,1,j]-y.diag[13,1,j]

y.diag[18,1,j] <- cut(tot.recent.NK[j])

y.diag[18,2,j] <- y.diag[11,1,j]-y.diag[18,1,j]

recent2009.IDU.RGA[j] <- sum(y.diag[13:17,1,j])

nrecent2009.IDU.RGA[j] <- sum(y.diag[13:17,2,j])

recent2009.NK.RGA[j] <- sum(y.diag[18:22,1,j])

nrecent2009.NK.RGA[j] <- sum(y.diag[18:22,2,j])

recent2009.RGA[j] <-recent2009.IDU.RGA[j]+recent2009.NK.RGA[j]

nrecent2009.RGA[j] <- nrecent2009.IDU.RGA[j]+nrecent2009.NK.RGA[j]

PWID2009.RGA[j] <- recent2009.RGA[j]+nrecent2009.RGA[j]

nonPWID2009.RGA[j] <- y.diag[3,2,j] + y.diag[4,2,j]

prop.recent.RGA[j] <- recent2009.RGA[j]/PWID2009.RGA[j]

uk.IDU[j] <- y.diag[4,1,j]

}

glas.recent <- sum(recent2009.RGA[1:4])

else.recent <- sum(recent2009.RGA[5:8])

glas.nrecent <- sum(nrecent2009.RGA[1:4])

else.nrecent <- sum(nrecent2009.RGA[5:8])

all.recent <- sum(recent2009.RGA[])

all.nrecent <- sum(nrecent2009.RGA[])

all.PWID <- all.recent+all.nrecent

all.nonPWID <- sum(nonPWID2009.RGA[])

prop.glas.recent <- glas.recent/(glas.recent+glas.nrecent)

prop.else.recent <- else.recent/(else.recent+else.nrecent)

prop.all.recent<- all.recent/(all.recent+all.nrecent)

tot.uk.IDU <- sum(uk.IDU[])

#-------------------------------

# Prior distributions

#-------------------------------

prob[1] ~ dbeta(1,1)

prob[2] ~ dbeta(1,1)T(lower.prob2,)

lower.prob2 <- prob[3]+0.001

prob[3] ~ dunif(0.6,upper.prob3)

upper.prob3 <- prob[2]-0.001

for(i in 4:11){

prob[i] ~ dbeta(1,1)

}

prob[14] ~ dbeta(1,1)T(lower.prob14,)

lower.prob14 <- prob[16]+0.001

prob[16] ~dbeta(1,1)T(,upper.prob16)

upper.prob16 <- prob[14]-0.001

prob[19] ~ dbeta(1,1)T(lower.prob19,)

lower.prob19 <- prob[21]+0.001

prob[21] ~ dbeta(1,1)T(,upper.prob21)

upper.prob21 <- prob[19]-0.001

prob[12] <-sum( y.diag[13,1,])/sum(y.diag[9,1,])

prob[17] <- sum(y.diag[18,1,])/sum(y.diag[11,1,])

alpha ~ dnorm(0,1.0E-4)

beta.inj[1] <- 0

for(i in 2:6){

beta.inj[i] ~ dnorm(0,1.0E-4)

}

beta.year[4] <- 0

for(i in 1:3){

beta.year[i] ~ dnorm(0,1.0E-4)

}

beta.age[1] <-0

for(i in 2:5){

beta.age[i] ~ dnorm(0,1.0E-4)

}

beta.diag[1] <-0

for(i in 2:3){

beta.diag[i] ~ dnorm(0,1.0E-4)

}

beta.gender ~ dnorm(0,1.0E-4)

beta.glasgow ~ dnorm(0,1.0E-4)

}

## Stage 2 : MPES model of HCV prevalence in Scotland combining NESI data,

## SHCDD and linked SDMD data and stage 1 model estimates.

## i = recent/non-recent PWID

## r = region(Glasgow/Elsewhere)

## g = gender(male/female),

## a = age(15-34,35-64)

## j = RGA (Region/Gender/Age)

## 1: Glasgow, male, <35

## 2: Glasgow, male, 35+

## 3: Glasgow, female, <35

## 4: Glasgow, female, 35+

## 5: Elsewhere, male, <35

## 6: Elsewhere, male, 35+

## 7: Elsewhere, female, <35

## 8: Elsewhere, female, 35+

model

{

#----------------------------------------------------------------------------------------

# NESI data - prevalence (pi) and proportion diagnosed (delta)

#----------------------------------------------------------------------------------------

for(i in 1:2){

for(r in 1:2){

for (g in 1:2){

for(a in 1:2){

y.pi[i,r,g,a] ~ dbin(pi[i,r,g,a], n.pi[i,r,g,a])

y.delta[i,r,g,a] ~ dbin(delta.bias[i,r,g,a], y.pi[i,r,g,a])

yhat.pi[i,r,g,a] <- n.pi[i,r,g,a]*pi[i,r,g,a]

yhat.delta[i,r,g,a] <- y.pi[i,r,g,a]*delta.bias[i,r,g,a]

dev.pi[i,r,g,a] <- 2*(y.pi[i,r,g,a]*(log(y.pi[i,r,g,a])–

log(yhat.pi[i,r,g,a]))+((n.pi[i,r,g,a]-y.pi[i,r,g,a])*

(log(n.pi[i,r,g,a]-y.pi[i,r,g,a])-log(n.pi[i,r,g,a]-

yhat.pi[i,r,g,a]))))

dev.delta[i,r,g,a] <- 2*(y.delta[i,r,g,a]*(log(y.delta[i,r,g,a])–

log(yhat.delta[i,r,g,a]))+((y.pi[i,r,g,a]-y.delta[i,r,g,a])*

(log(y.pi[i,r,g,a]-y.delta[i,r,g,a])-log(y.pi[i,r,g,a]–

yhat.delta[i,r,g,a]))))

#---------------------------------------------------------------------

# Bias model for delta

#---------------------------------------------------------------------

logit(delta.bias[i,r,g,a]) <- logit(delta[i,r,g,a])+bias.delta[a]

}

}

}

}

#------------------------------------------------------------------------------------------------------------

# CRC data - population proportions of recent PWID (by region, gender, age)

# (using posteriors as priors for rho)

#------------------------------------------------------------------------------------------------------------

for(j in 1:8){

bin[j] ~ dcat(p.rho.CRC[j,1:n.bin[j]])

CRC.rho[j] <- rho.CRC.bin[j,bin[j]]

}

rho[1,1,1,1] <- CRC.rho[1]; rho[1,1,1,2] <- CRC.rho[2]; rho[1,1,2,1] <- CRC.rho[3]

rho[1,1,2,2] <- CRC.rho[4]; rho[1,2,1,1] <- CRC.rho[5]; rho[1,2,1,2] <- CRC.rho[6]

rho[1,2,2,1] <- CRC.rho[7]; rho[1,2,2,2] <- CRC.rho[8]

#-------------------------------------------------------------------------------------------------------------------------------------

# SHCDD and linked SDMD data :

# y.diag= posterior estimate of number of diagnosed recent PWID and all PWID from stage 1 model

# sd.diag = posterior std dev of number of diagnosed recent PWID and all PWID from stage 1 model

#-------------------------------------------------------------------------------------------------------------------------------------

for(i in 1:2){

for(r in 1:2) {

for(g in 1:2){

for(a in 1:2){

y.diag[i,r,g,a] ~ dnorm(SHCDD.total.diag[i,r,g,a],

tau.diag[i,r,g,a])

tau.diag[i,r,g,a] <- 1/(sd.diag[i,r,g,a]*sd.diag[i,r,g,a])

dev.diag[i,r,g,a] <- pow((y.diag[i,r,g,a]-

SHCDD.total.diag[i,r,g,a]),2)*tau.diag[i,r,g,a]

}

}

}

}

#-------------------------------------------------------------------------------------------------------------------------------------

# Bias parameter for number of diagnosed recent PWID in SHCDD and linked SDMD data

#-------------------------------------------------------------------------------------------------------------------------------------

for(r in 1:2){

for(g in 1:2){

for(a in 1:2){

log(SHCDD.total.diag[1,r,g,a]) <- log(diag.RGA[1,r,g,a])+log.bias.d[a]

log(SHCDD.total.diag[2,r,g,a]) <- log(diag.RGA[1,r,g,a]+

diag.RGA[2,r,g,a])

}

}

}

#--------------------------------------------------------------------------------------------------------------------

# Calculate estimates by region, gender & age

#--------------------------------------------------------------------------------------------------------------------

for(i in 1:2){

for(r in 1:2) {

for(g in 1:2){

for(a in 1:2){

prev.RGA[i,r,g,a] <- pop[r,g,a]*rho[i,r,g,a]*pi[i,r,g,a]

diag.RGA[i,r,g,a] <- pop[r,g,a]*rho[i,r,g,a]*pi[i,r,g,a]*

delta[i,r,g,a]

undiag.RGA[i,r,g,a] <- pop[r,g,a]*rho[i,r,g,a]*pi[i,r,g,a]*

(1-delta[i,r,g,a])

pop.risk.RGA[i,r,g,a] <- pop[r,g,a]*rho[i,r,g,a]

weight.pi.RGA[i,r,g,a] <- pi[i,r,g,a]*pop.risk.RGA[i,r,g,a]

weight.delta.RGA[i,r,g,a] <- delta[i,r,g,a]*pop.risk.RGA[i,r,g,a]

}

}

}

}

#----------------------------------------------------------

# Estimates by recent/non-recent PWID

#----------------------------------------------------------

for(i in 1:2){

prev.PWID[i] <- sum(prev.RGA[i,,,])

diag.PWID[i] <- sum(diag.RGA[i,,,])

undiag.PWID[i] <- sum(undiag.RGA[i,,,])

pop.risk.PWID[i] <- sum(pop.risk.RGA[i,,,])

rho.PWID[i] <- pop.risk.PWID[i]/sum(pop[,,])

pi.PWID[i] <- sum(weight.pi.RGA[i,,,])/pop.risk.PWID[i]

delta.PWID[i] <- sum(weight.delta.RGA[i,,,])/pop.risk.PWID[i]

percent.risk.PWID[i] <- pop.risk.PWID[i]/sum(pop.risk.PWID[])

#--------------------------------------------------------------------------

# Estimates by recent/non-recent PWID and region

#--------------------------------------------------------------------------

for(r in 1:2){

regPWID.risk[i,r] <- pop.risk.RGA[i,r,1,1]+pop.risk.RGA[i,r,1,2]+

pop.risk.RGA[i,r,2,1]+pop.risk.RGA[i,r,2,2]

regPWID.prev[i,r] <- prev.RGA[i,r,1,1]+prev.RGA[i,r,1,2]+prev.RGA[i,r,2,1]+

prev.RGA[i,r,2,2]

regPWID.diag[i,r] <- diag.RGA[i,r,1,1]+diag.RGA[i,r,1,2]+diag.RGA[i,r,2,1]+

diag.RGA[i,r,2,2]

regPWID.undiag[i,r] <- undiag.RGA[i,r,1,1]+undiag.RGA[i,r,1,2]+

undiag.RGA[i,r,2,1]+undiag.RGA[i,r,2,2]

regPWID.rho[i,r] <- regPWID.risk[i,r]/(pop[r,1,1]+pop[r,1,2]+

pop[r,2,1]+pop[r,2,2])

regPWID.pi[i,r] <- (weight.pi.RGA[i,r,1,1]+weight.pi.RGA[i,r,1,2]+

weight.pi.RGA[i,r,2,1]+weight.pi.RGA[i,r,2,2])/

regPWID.risk[i,r]

regPWID.delta[i,r] <- (weight.delta.RGA[i,r,1,1]+weight.delta.RGA[i,r,1,2]+

weight.delta.RGA[i,r,2,1]+weight.delta.RGA[i,r,2,2])/

regPWID.risk[i,r]

}

}

#-------------------------------------

# Estimates by gender

#------------------------------------

for(g in 1:2){

gender.prev[g] <- prev.RGA[1,1,g,1]+prev.RGA[1,1,g,2]+prev.RGA[1,2,g,1]

+prev.RGA[1,2,g,2]+prev.RGA[2,1,g,1]+prev.RGA[2,1,g,2]

+prev.RGA[2,2,g,1]+prev.RGA[2,2,g,2]

gender.undiag[g] <- undiag.RGA[1,1,g,1]+undiag.RGA[1,1,g,2]+undiag.RGA[1,2,g,1]

+undiag.RGA[1,2,g,2]+undiag.RGA[2,1,g,1]+undiag.RGA[2,1,g,2]

+undiag.RGA[2,2,g,1]+undiag.RGA[2,2,g,2]

gender.diag[g] <- diag.RGA[1,1,g,1]+diag.RGA[1,1,g,2]+diag.RGA[1,2,g,1]

+diag.RGA[1,2,g,2]+diag.RGA[2,1,g,1]+diag.RGA[2,1,g,2]

+diag.RGA[2,2,g,1]+diag.RGA[2,2,g,2]

gender.risk[g] <- pop.risk.RGA[1,1,g,1]+pop.risk.RGA[1,1,g,2]+pop.risk.RGA[1,2,g,1]

+pop.risk.RGA[1,2,g,2]+pop.risk.RGA[2,1,g,1]+pop.risk.RGA[2,1,g,2]

+pop.risk.RGA[2,2,g,1]+pop.risk.RGA[2,2,g,2]

gender.rho[g] <- gender.risk[g]/(pop[1,g,1]+pop[1,g,2]+pop[2,g,1]+pop[2,g,2])

gender.pi[g] <- (weight.pi.RGA[1,1,g,1]+weight.pi.RGA[1,1,g,2]+

weight.pi.RGA[1,2,g,1]+weight.pi.RGA[1,2,g,2]+

weight.pi.RGA[2,1,g,1]+weight.pi.RGA[2,1,g,2]+ weight.pi.RGA[2,2,g,1]+weight.pi.RGA[2,2,g,2])/gender.risk[g]

gender.delta[g] <- (weight.delta.RGA[1,1,g,1]+weight.delta.RGA[1,1,g,2]+

weight.delta.RGA[1,2,g,1]+weight.delta.RGA[1,2,g,2]+

weight.delta.RGA[2,1,g,1]+weight.delta.RGA[2,1,g,2]+

weight.delta.RGA[2,2,g,1]+weight.delta.RGA[2,2,g,2])/

gender.risk[g]

}

#-------------------------------------

# Estimates by region

#------------------------------------

for(r in 1:2){

region.prev[r] <- prev.RGA[1,r,1,1]+prev.RGA[1,r,1,2]+prev.RGA[1,r,2,1]+

prev.RGA[1,r,2,2]+prev.RGA[2,r,1,1]+prev.RGA[2,r,1,2]+

prev.RGA[2,r,2,1]+prev.RGA[2,r,2,2]

region.diag[r] <- diag.RGA[1,r,1,1]+diag.RGA[1,r,1,2]+diag.RGA[1,r,2,1]+

diag.RGA[1,r,2,2]+diag.RGA[2,r,1,1]+diag.RGA[2,r,1,2]+

diag.RGA[2,r,2,1]+diag.RGA[2,r,2,2]

region.undiag[r] <- undiag.RGA[1,r,1,1]+undiag.RGA[1,r,1,2]+undiag.RGA[1,r,2,1]+

undiag.RGA[1,r,2,2]+undiag.RGA[2,r,1,1]+undiag.RGA[2,r,1,2]+

undiag.RGA[2,r,2,1]+undiag.RGA[2,r,2,2]

region.risk[r] <- pop.risk.RGA[1,r,1,1]+pop.risk.RGA[1,r,1,2]+pop.risk.RGA[1,r,2,1]+

pop.risk.RGA[1,r,2,2]+pop.risk.RGA[2,r,1,1]+pop.risk.RGA[2,r,1,2]+

pop.risk.RGA[2,r,2,1]+pop.risk.RGA[2,r,2,2]

region.rho[r] <- region.risk[r]/(pop[r,1,1]+pop[r,1,2]+pop[r,2,1]+pop[r,2,2])

region.pi[r] <- (weight.pi.RGA[1,r,1,1]+weight.pi.RGA[1,r,1,2]+weight.pi.RGA[1,r,2,1]+

weight.pi.RGA[1,r,2,2]+weight.pi.RGA[2,r,1,1]+weight.pi.RGA[2,r,1,2]+

weight.pi.RGA[2,r,2,1]+weight.pi.RGA[2,r,2,2])/region.risk[r]

region.delta[r] <- (weight.delta.RGA[1,r,1,1]+weight.delta.RGA[1,r,1,2]+

weight.delta.RGA[1,r,2,1]+weight.delta.RGA[1,r,2,2]+

weight.delta.RGA[2,r,1,1]+weight.delta.RGA[2,r,1,2]+

weight.delta.RGA[2,r,2,1]+weight.delta.RGA[2,r,2,2])/region.risk[r]

}

#-------------------------------------

# Estimates by age

#------------------------------------

for(a in 1:2){

age.prev[a] <- prev.RGA[1,1,1,a]+prev.RGA[1,1,2,a]+prev.RGA[1,2,1,a]+

prev.RGA[1,2,2,a]+prev.RGA[2,1,1,a]+prev.RGA[2,1,2,a]+

prev.RGA[2,2,1,a]+prev.RGA[2,2,2,a]

age.diag[a] <- diag.RGA[1,1,1,a]+diag.RGA[1,1,2,a]+diag.RGA[1,2,1,a]+

diag.RGA[1,2,2,a]+diag.RGA[2,1,1,a]+diag.RGA[2,1,2,a]+

diag.RGA[2,2,1,a]+diag.RGA[2,2,2,a]

age.undiag[a] <- undiag.RGA[1,1,1,a]+undiag.RGA[1,1,2,a]+undiag.RGA[1,2,1,a]+

undiag.RGA[1,2,2,a]+undiag.RGA[2,1,1,a]+undiag.RGA[2,1,2,a]+

undiag.RGA[2,2,1,a]+undiag.RGA[2,2,2,a]

age.risk[a] <- pop.risk.RGA[1,1,1,a]+pop.risk.RGA[1,1,2,a]+pop.risk.RGA[1,2,1,a]+

pop.risk.RGA[1,2,2,a]+pop.risk.RGA[2,1,1,a]+pop.risk.RGA[2,1,2,a]+

pop.risk.RGA[2,2,1,a]+pop.risk.RGA[2,2,2,a]

age.rho[a] <- age.risk[a]/(pop[1,1,a]+pop[1,2,a]+pop[2,1,a]+pop[2,2,a])

age.pi[a] <- (weight.pi.RGA[1,1,1,a]+weight.pi.RGA[1,1,2,a]+weight.pi.RGA[1,2,1,a]+

weight.pi.RGA[1,2,2,a]+weight.pi.RGA[2,1,1,a]+weight.pi.RGA[2,1,2,a]+

weight.pi.RGA[2,2,1,a]+weight.pi.RGA[2,2,2,a])/age.risk[a]

age.delta[a] <- (weight.delta.RGA[1,1,1,a]+weight.delta.RGA[1,1,2,a]+

weight.delta.RGA[1,2,1,a]+weight.delta.RGA[1,2,2,a]+

weight.delta.RGA[2,1,1,a]+weight.delta.RGA[2,1,2,a]+

weight.delta.RGA[2,2,1,a]+weight.delta.RGA[2,2,2,a])/age.risk[a]

age.undiag.pc[a] <- age.undiag[a]/sum(age.undiag[])

}

all.prev <- sum(prev.PWID[])

all.diag <- sum(diag.PWID[])

all.undiag <- sum(undiag.PWID[])

all.risk <- sum(pop.risk.PWID[])

all.rho <- all.risk/sum(pop[,,])

all.pi <- sum(weight.pi.RGA[,,,])/all.risk

all.delta <- sum(weight.delta.RGA[,,,])/all.risk

percent.recent <- pop.risk.PWID[1]/all.risk

percent.undiag <- all.undiag/all.prev

percent.undiag.NR <- undiag.PWID[2]/all.undiag

sum.dev <- sum(dev.pi[,,,])+sum(dev.delta[,,,])+sum(dev.diag[,,,])

#-------------------------------------------------------------------------------------------------------

# Priors (bias parameters bounded by log(0.5) & log(5) and age ordered)

#-------------------------------------------------------------------------------------------------------

log.bias.d[1] ~ dnorm(0,1.0E-4)I(-0.69,log.bias.d[2])

log.bias.d[2] ~ dnorm(0,1.0E-4)I(log.bias.d[1],1.61)

bias.delta[1] ~ dnorm(0,1.0E-4)I(-0.69,bias.delta[2])

bias.delta[2] ~ dnorm(0,1.0E-4)I(bias.delta[1],1.61)

for(a in 1:2){

exp.bias.delta[a] <- exp(bias.delta[a])

bias.d[a] <- exp(log.bias.d[a])

}

for(r in 1:2){

for(g in 1:2){

for(a in 1:2){

for (i in 1:2) {

pi[i,r,g,a] ~ dbeta(1,1)

delta[i,r,g,a] ~ dbeta(1,1)

}

rho[2,r,g,a] ~ dbeta(1,1)

}

}

}

}
